# Outcomes of emergent cardiac surgery after transcatheter aortic valve implantation

**DOI:** 10.1007/s12471-023-01820-0

**Published:** 2023-11-02

**Authors:** Gijs J. van Steenbergen, Jules R. Olsthoorn, Rob Eerdekens, Erwin Tan, Pim A. L. Tonino, Ka Yan Lam

**Affiliations:** 1 Department of Cardiothoracic Surgery, Catharina Heart Centre, Catharina Hospital, Eindhoven, The Netherlands; 2 Department of Cardiology, Catharina Heart Centre, Catharina Hospital, Eindhoven, The Netherlands

**Keywords:** Aortic valve replacement, TAVI, Cardiac surgery, AVR

## Abstract

***Objective*:**

The aim of this study was to evaluate the reasons for emergent cardiac surgery (ECS) after transcatheter aortic valve implantation (TAVI) and assess outcomes of these patients.

***Methods*:**

All patients undergoing ECS following a complicated TAVI procedure at a high-volume TAVI centre in the Netherlands from 1 January 2008 to 1 April 2022 were included. Baseline and procedural characteristics and outcome data (procedural, 30-day and 1‑year mortality, in-hospital stroke, 30-day pacemaker implantation, 30-day vascular complications, 30-day deep sternal wound infections and 30-day re-exploration) were collected from patient files and analysed using descriptive statistics.

**Results:**

During the study period, 16 of 1594 patients (1.0%) undergoing TAVI required ECS. The main reason for ECS was valve embolisation (*n* = 9; 56.3%), followed by perforation of the left/right ventricle with guide wire/pacemaker lead (*n* = 3; 18.8%) and annular rupture (*n* = 3; 18.8%). Procedural, 30-day and 1‑year mortality was 0%, 18.8% (*n* = 3) and 31.3% (*n* = 5), respectively. In-hospital stroke occurred in 1 patient (6.3%), a pacemaker was implanted at 30 days in 2 patients (12.5%), and major vascular complications did not occur.

***Conclusion*:**

ECS following complicated TAVI was performed in only a small number of cases. It had a high but acceptable perioperative and 30-day mortality, taking into account the otherwise lethal consequences. In case of valve embolisation, no periprocedural or 30-day mortality was observed for surgical aortic valve replacement (even in a redo setting), which supported the necessity to perform TAVI in centres with cardiac surgical backup on site.

## What’s new?

**Fig. 1:**
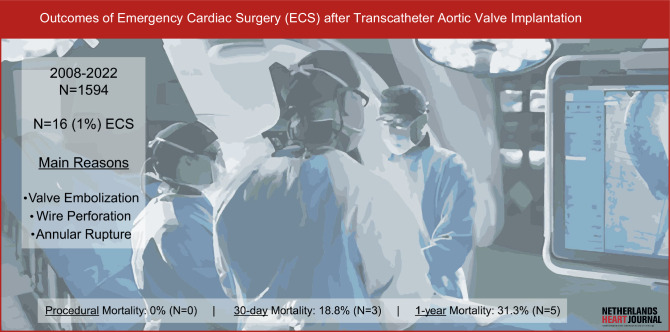
Infographic

- From 1 January 2008 to 1 April 2022, 1.0% of the patients undergoing transcatheter aortic valve implantation (TAVI) at a Dutch high-volume TAVI centre required emergent cardiac surgery.
- In case of valve embolisation, no periprocedural or 30-day mortality was observed (Fig. 1).

## Introduction

Aortic valve stenosis is one of the most prevalent valvular conditions in Europe and North-America, affecting roughly 12% of the population > 75 years of age [1]. In symptomatic patients with severe aortic stenosis, aortic valve replacement is the treatment of choice, and surgical aortic valve replacement (SAVR) is the gold standard. Transcatheter aortic valve implantation (TAVI) was introduced in the early 2000s and has been shown to be safe and effective in several randomised trials and real-world data analyses for multiple patients groups [2–4]. The choice for SAVR or TAVI is based on patient and anatomical characteristics, as well as current guideline recommendations, and is made by the Heart Team, consisting of at least one cardiothoracic surgeon and one cardiologist.

Although TAVI is less invasive than SAVR, it carries the potential risk of some rare complications, such as annular rupture, ventricular perforation or embolisation of the valve prosthesis, that may ultimately require emergent cardiac surgery (ECS). Hence, it is recommended that TAVI procedures are performed using immediate cardiopulmonary bypass and with surgical backup [5]. Although conversion to sternotomy is not common, the mortality of ECS can be as high as 70% in some studies, depending on the nature of the underlying complication [6–8]. Studies on adverse events during follow-up other than mortality are very scarce, and these adverse events therefore constitute an important knowledge hiatus. The European Registry on Emergent Cardiac Surgery during TAVI (EuRECS-TAVI), real-world, multicentre registry, provides some insight but also has a strong focus on mortality [9].

In the current study, we therefore assessed the incidence of ECS following complicated TAVI at a high-volume TAVI centre in the Netherlands, with a special focus on the reasons for surgery and postoperative outcomes.

## Methods

### Study setting and design

This was a retrospective cohort study of consecutive patients conducted at the Catharina Heart Centre of the Catharina Hospital in Eindhoven, the Netherlands. The PROCESS checklist was used as a reporting guideline [10]. The study was performed in accordance with the Declaration of Helsinki and was approved by the local ethics committee, who waived the need for informed consent.

### Study population

All patients who underwent TAVI at our centre from 1 January 2008 to 1 April 2022 were screened. Patients who underwent ECS as a result of a TAVI complication were included for in-depth analysis. Only patients who underwent cardiac surgery during the same admission as the TAVI procedure were included. Patients who underwent elective cardiac surgery after TAVI for any reason were excluded.

### Data collection and definitions

For the included patients, the following baseline characteristics were retrospectively collected from patient files: age, gender, body mass index (BMI), presence or absence of diabetes mellitus, chronic pulmonary disease, peripheral vascular disease and renal impairment (glomerular filtration rate according to Modification of Diet in Renal Disease < 60 ml/min per 1.73 m^2^), previous stroke, myocardial infarction in the last 90 days (as defined by Thygesen et al. [11]), previous cardiac surgery and previous pacemaker implantation, left ventricular ejection fraction (LVEF), pulmonary artery pressure, New York Heart Association (NYHA) class, and logistic European System for Cardiac Operative Risk Evaluation (EuroSCORE) and EuroSCORE II as proposed by Roques et al. [12] Baseline characteristics were defined in accordance with the Netherlands Heart Registry (*Nederlandse Hart Registratie*, NHR) unless stated otherwise [13].

Furthermore, the following procedural parameters were retrospectively collected: reason for TAVI, access route of TAVI, valve type used (a distinction was made between self-expandable valves (Evolut R, Pro+ and CoreValve; Medtronic Inc., Minneapolis, MN, USA), balloon-expendable valves (Sapien 3 and XT; Edwards Lifesciences, Irvine, CA, USA) and valves used for study purposes (Direct Flow Medical, Inc., Santa Rosa, CA, USA)), reason for sternotomy/cardiac surgery, type of surgery performed and use of cardiopulmonary bypass.

Primary outcomes were procedural, 30-day and 1‑year mortality. Secondary outcomes included in-hospital stroke, 30-day pacemaker implantation, 30-day vascular complications, 30-day deep sternal wound infections and 30-day re-exploration. These parameters were defined in accordance with the Valve Academic Research Consortium‑2 consensus document [14]. In our hospital, patients are actively followed up to provide these outcome variables to the NHR. Data from this follow-up were also used for the present study. Telephone follow-up was performed in case of missing outcome data.

At our hospital, continuous quality monitoring is part of standard care for cardiologic and cardiothoracic procedures, and, as such, TAVI procedures and cardiac surgeries were performed according to the standard of care at the time [15].

### Statistical analysis

General descriptive statistics were used to describe the patient characteristics. These characteristics are reported as median with interquartile range (IQR) for continuous non-normally distributed data and absolute and relative frequency for categorical data. All analyses were performed using SPSS 25 (IBM, Chicago, IL, USA).

## Results

The first TAVI procedure at our centre was performed in 2008. In the 13 years up to 1 April 2022, a total of 1594 TAVI procedures were performed. During this period, 16 patients (1.0%) required ECS after TAVI during the same admission and were therefore included in the analysis. ECS was less frequently performed during the early years (2008–2015; 9/333 (2.7%)) compared with later years (2016–2022; 8/1261 (0.63%)). Up to September 2018, 18 patients died during the TAVI procedure, compared with 6 patients in the period October 2018–April 2022. For reference, the procedural mortality (within 72 h) of the population without ECS at our facility during the same period was 1.5% (*n* = 24).

### Patient characteristics

The median age of the 16 ECS patients was 80 years (IQR: 78–86), and 12 of them (75.0%) were male. The majority (*n* = 11; 68.8%) experienced symptoms of NYHA class > II prior to TAVI. Three patients (18.8%) had previous cardiac surgery and 1 patient (6.3%) had previous aortic valve surgery. Median LVEF was 55% (IQR: 53–55), and the median EuroSCORE II was 3.25 (IQR: 1.55–8.47). Other baseline characteristics are shown in Tab. 1.

**Table 1:** Baseline characteristics of patients undergoing emergent cardiac surgery after TAVI and procedural data

Variable	Patients (*n* = 16)
**Patient characteristics**	
Age, years	80 (78–86)
Male	12 (75.0)
BMI, kg/m^2^	26.8 (22.8–31.2)
*Medical history*	
– Diabetes mellitus	6 (37.5)
– Chronic pulmonary disease	4 (25.0)
– Peripheral vascular disease	4 (25.0)
– Renal impairment^a^	2 (12.5)
– Previous stroke	2 (12.5)
– Recent MI (90 days)	1 (6.3)
– Previous cardiac surgery	3 (18.8)
– Previous aortic valve surgery	1 (6.3)
– Previous pacemaker implantation	2 (12.5)
LVEF, %	55 (53–55)
Pulmonary artery pressure, mmHg	25 (25–26)
NYHA class > II	11 (68.8)
*EuroSCORE*	
– Logistic	8.9 (5.5–14.5)
– II	3.3 (1.6–8.5)
**Procedural data**	
*Type of implanted valve*^b^	
– Self-expandable valve	7 (46.7)
– Balloon-expandable valve	7 (46.7)
– Direct Flow Medical valve	1 (6.7)
*Access route*	
– Femoral	14 (87.5)
– Apical	1 (6.3)
– Ascending aorta	1 (6.3)

Data are median (interquartile range) or *n* (%)

*TAVI* transcatheter aortic valve implantation, *BMI* body mass index, *MI* myocardial infarction, *LVEF* left ventricular ejection fraction, *NYHA* New York Heart Association

^a^ Glomerular filtration rate according to Modification of Diet in Renal Disease < 60 ml/min per 1.73 m^2^

^b^ *N* = 15 (no valve implanted in 1 patient)

### Procedural parameters

The main reason patients underwent TAVI instead of SAVR was high surgical risk, followed by inoperability (previous cardiac surgery with graft crossing sternal midline or calcified aorta). In some cases, patients requested TAVI instead of SAVR. The femoral artery was the most common access route for TAVI (*n* = 14; 87.5%) (Tab. 1). A self-expendable valve was used in 46.7% (7/15) of the patients, a balloon-expandable valve in 46.7% (7/15) and the Direct Flow Medical valve in 6.3%(1/15). In 1 patient, no valve was placed.

### TAVI complications

**Fig. 2:**
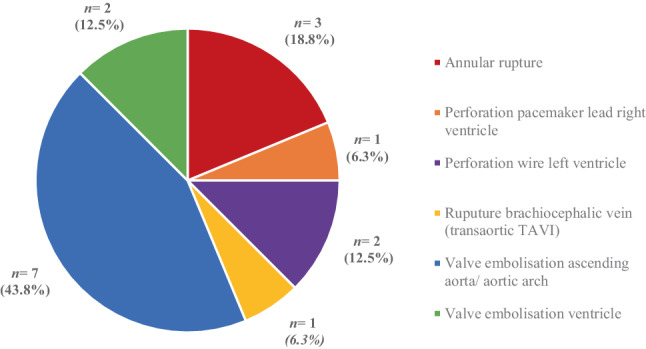
Reasons for emergent cardiac surgery after transcatheter aortic valve implantation (*TAVI*)

**Fig. 3:**
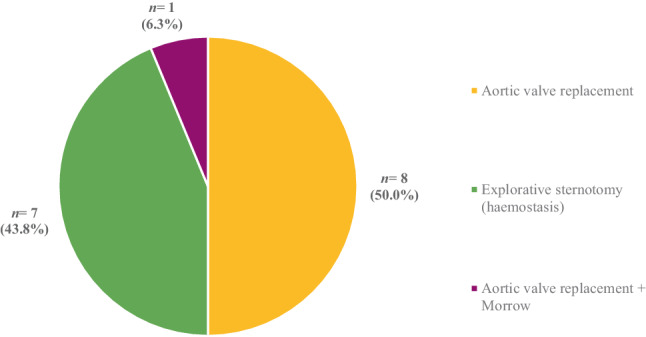
Types of surgery performed

Valve embolisation was the most prevalent reason for ECS during the study period (*n* = 9; 56.3%), followed by annular rupture (*n* = 3; 18.8%) and perforation of the left ventricle by guide wire (*n* = 2; 12.5%) (Fig. 2). Other reasons for cardiac surgery were malposition of the valve, bleeding other than annular rupture and perforation of the right ventricle by the pacemaker lead. Conventional AVR (*n* = 8; 50.0%) and explorative sternotomy (*n* = 7; 43.8%) were the most frequently performed cardiac surgeries during admission (Fig. 3). In 9 patients (56.3%), cardiopulmonary bypass was used (Tab. 2).

**Table 2:** Emergent cardiac surgery following complicated TAVI (*n* = 16)

Patient no.	Age, years	EuroSCORE		Reason for TAVI over SAVR	Access	Valve type	Reason for sternotomy	Type of surgery	CPB	30-day mortality
		Logistic	II							
1	87	8.99	1.44	Surgical risk	Femoral	Self-expandable	Perforation pacemaker lead RV	Explorative sternotomy	No	No
2	80	5.48	2.65	Calcified aorta	Apex	Balloon-expandable	Valve embolisation	SAVR	Yes	No
3	75	17.9	10.79	Surgical risk (previous CABG)	Aorta	NA	Rupture brachiocephalic vein	Explorative sternotomy	No	Yes
4	87	14.49	9.12	Palliative care	Femoral	Balloon-expandable	Annular rupture	Explorative sternotomy	No	No
5	79	8.10	3.25	Surgical risk	Femoral	Direct Flow Medical	Valve embolisation	SAVR + Morrow	Yes	No
6	86	9.79	8.08	Patient request	Femoral	Balloon-expandable	Perforation wire LV	Explorative sternotomy	No	Yes
7	85	4.49	4.59	Surgical risk	Femoral	Self-expandable	Valve embolisation	SAVR	Yes	No
8	50	5.48	15.85	Bridge to SAVR	Femoral	Self-expandable	Valve embolisation	SAVR	Yes	No
9	79	20.42	1.72	Surgical risk	Femoral	Self-expandable	Valve embolisation	SAVR	Yes	No
10	79	7.46	1.44	Surgical risk	Femoral	Balloon-expandable	Perforation wire LV	Explorative sternotomy	No	No
11	81	9.15	1.55	Palliative care	Femoral	Balloon-expandable	Valve embolisation	SAVR	Yes	No
12	83	21.30	15.71	Neurological condition	Femoral	Self-expandable	Valve embolisation	SAVR	Yes	No
13	77	4.25	1.04	Surgical risk	Femoral	Self-expandable	Valve embolisation	SAVR	Yes	No
14	78	7.46	3.67	Anatomy	Femoral	Balloon-expandable	Annular rupture	Explorative sternotomy	No	No
15	88	8.99	2.44	Surgical risk	Femoral	Balloon-expandable	Annular rupture	Explorative sternotomy	No	Yes
16	85	14.61	8.47	Surgical risk	Femoral	Balloon-expandable	Valve embolisation	SAVR	Yes	No

*TAVI* transcatheter aortic valve implantation, *SAVR* surgical aortic valve replacement, *CPB* cardiopulmonary bypass, *RV* right ventricle, *CABG* coronary artery bypass grafting, *NA* not applicable, *LV* left ventricle

Four patients received TAVI under local anaesthesia. Three of them had complications that prevented them from deciding whether or not to proceed due to instability or resuscitation. One patient with a stable haemodynamic condition gave informed consent for SAVR after TAVI valve dislocation. In the remaining patients, the decision to operate was made by the cardiologist and cardiac surgeon based on the complication type and the rationale for TAVI over SAVR.

### Clinical outcomes

No patients died during the surgical procedure. The 30-day mortality was 18.8% (*n* = 3), whereas the 1‑year mortality was 31.3% (*n* = 5) (Tab. 3). Causes of mortality were sudden cardiac death (*n* = 3) and refractory heart failure (*n* = 2). In-hospital stroke occurred in 1 patient (6.3%). Two patients (12.5%) required pacemaker implantation within 30 days. Re-exploration was necessary in 2 patients (12.5%), once for bleeding and once for refixation of the sternum. Within 30 days after ECS, there were no major vascular complications or deep sternal wound infections.

**Table 3:** Clinical outcomes after ECS following complicated TAVI

Outcome	Patients (*n* = 16)
Procedural mortality	0
30-day mortality	3 (18.8)
1‑year mortality	5 (31.3)
In-hospital stroke	1 (6.3)
30-day pacemaker implantation	2 (12.5)
30-day re-exploration	2 (12.5)
30-day deep sternal wound infections	0
30-day major vascular complications after ECS	0

Data are *n* (%)

*ECS* emergent cardiac surgery, *TAVI* transcatheter aortic valve implantation

## Discussion

The principal finding of our study was that although ECS was not frequently performed after TAVI, it was associated with high mortality at 30 days and 1 year. One-third of patients had died at 1 year follow-up, even though procedural mortality was 0. In addition, the causes for mid-term mortality were not directly related to the initial TAVI-related complication or the ECS performed. Based on our findings, ECS after complicated TAVI should therefore not be omitted when deemed necessary.

These findings are in corroboration with those from previous studies. In the largest meta-analysis to date, including over 9000 patients from 46 studies, ECS after complicated TAVI was performed in 1.1% of cases—in two-thirds of patients, the femoral access route was used [5]. A more recent analysis of data from the EuRECS-TAVI, comprising 27,760 patients from 79 centres, showed an incidence of ECS after transfemoral TAVI of 0.76% [9]. Similar to our study results, embolisation and valve dislocation, guidewire perforation and annular rupture were the most common reasons for ECS in both studies [5, 9]. Patient profiles were also comparable to our study population, with a mean logistic EuroSCORE of ~20 (24 and 17, respectively) and mean age of ~82 years (81.3 and 82.4 years, respectively).

Even though TAVI is considered safe and effective in patients with a high, intermediate and low risk of operative mortality in multiple trials, in current clinical practice, those selected for TAVI are still usually elderly, frail patients with comorbidities who are deemed either inoperable or at high risk for SAVR [16]. The occurrence of a complication during TAVI for which sternotomy is deemed necessary therefore raises a potential ethical dilemma. Conducting ECS for complicated TAVI should be carefully considered in a multidisciplinary discussion with patients (if the clinical situation at the time permits this), cardiologists and surgeons, preferably prior to the procedure.

Our findings indicate that ECS after complicated TAVI can be performed safely, although it has a high but acceptable perioperative and 30-day mortality, taking into account the otherwise potentially lethal consequences. Nevertheless, the suspected nature of the complication should be taken into account as there are some studies describing a variety of outcomes depending on the type of complication (e.g. [15]). Event rates in our study were too low to make a distinction per type of complication, but the EuRECS-TAVI suggests that mortality of, for example, valve embolisation is ~22% and that of annular rupture is ~62% [9].

The decision to perform ECS after a complicated TAVI procedure should therefore also be driven by the nature of the complication itself. On the contrary, if the patient is not treated with ECS, the 30-day mortality may exceed the 18.8% found in this study. Furthermore, the 9 patients requiring ECS for valve embolisation had no procedural and 30-day mortality and therefore support the strategy to perform ECS despite comorbidities. In the TAVI population at our facility that did not undergo ECS during the same period, the cause of death in only 1 patient was valve prosthesis dislocation, thus supporting the necessity to perform TAVI in centres with cardiac surgical backup on site.

To our knowledge, the current study is the first to report complications other than mortality after ECS for complicated TAVI. In our cohort, 2/16 patients (12.5%) had to undergo re-exploration, which exceeds the average incidence for cardiac surgery in the Netherlands (~5%) [17]. However, this high re-exploration rate in our study did not come with an increased incidence of deep sternal wound infections, even though multiple procedures are a known risk factor for this complication [18]. The rate of permanent pacemaker implantation after ECS was also higher than the national average (~5%), which was most likely attributable to the preceding TAVI procedure as permanent pacemaker implantation after TAVI in general is estimated to be 14% [17–19].

### Limitations

This retrospective study had limited power to draw firm conclusions due to the low frequency of complications after ECS. Additionally, relevant parameters such as predictors of ECS and causality analysis could not be identified due to an insufficient number of events. Furthermore, the operator’s expertise and experience level may impact ECS after TAVI, although the EuRECS-TAVI found no difference in mortality between low- and high-volume centres [9]. Finally, data on long-term functional status and quality of life were not available.

## Conclusion

ECS following complicated TAVI occurs at a low frequency. ECS after complicated TAVI has a high but acceptable perioperative and 30-day mortality, taking into account the otherwise potential lethal consequences. In case of valve embolisation, no periprocedural or 30-day mortality was observed for SAVR (even in a redo setting), which supports the necessity to perform TAVI in centres with cardiac surgical backup on site. ECS after complicated TAVI should be considered by a Heart Team prior to the procedure.
